# Detection of *Clostridium perfringens* Using Novel Methods Based on Recombinase-Aided Amplification Assay-Assisted CRISPR/Cas12a System

**DOI:** 10.1155/2023/6667618

**Published:** 2023-11-14

**Authors:** Xingxing Xiao, Qingxun Zhang, Sihong Wu, Yi Li, Zhenyu Zhong, Qingyun Guo, Junfang Li, Qinghui Meng, Zhibin Cheng, Jianbin Duan, Xiaoqiong Wang, Hongxuan He, Jiade Bai, Yongliang Lou

**Affiliations:** ^1^Wenzhou Key Laboratory of Sanitary Microbiology, Key Laboratory of Laboratory Medicine, Ministry of Education, School of Laboratory Medicine and Life Sciences, Wenzhou Medical University, Wenzhou 325035, China; ^2^Beijing Milu Ecological Research Center, Beijing Academy of Science and Technology, Beijing 100076, China; ^3^National Research Center for Wildlife-Born Diseases, Institute of Zoology, Chinese Academy of Sciences, Beijing 100101, China; ^4^Zhuji Institute of Biomedicine, Wenzhou Medical University, Zhuji, Shaoxing, Zhejiang 311800, China

## Abstract

*Clostridium perfringens* is a highly versatile pathogen of humans and animals. Rapid and sensitive detection methods for *C. perfringens* are urgently needed for the timely implementation of control. In this study, to provide novel promising methods for the detection of *C. perfringens*, two rapid, sensitive, and instrument-free *C. perfringens* detection methods based on recombinase-aided amplification (RAA) assay and clustered regularly interspaced short palindromic repeats (CRISPR) and CRISPR-associated protein 12a (CRISPR/Cas12a) system were developed depending on fluorescence signal (RAA-CRISPR/Cas12a-FL) and lateral flow strip (RAA-CRISPR/Cas12a-LFS), respectively. The limit of detection of the RAA-CRISPR/Cas12a-FL and RAA-CRISPR/Cas12a-LFS methods is 2 copies and 20 copies of *C. perfringens* genomic DNA per reaction, respectively, and the whole process can be completed in 1 hr. Moreover, these two methods show no cross-reactivity with nontarget bacteria, which were used as a negative control to evaluate the specificity of two developed methods in the detection of *C. perfringens* and have 100% consistent with real-time polymerase chain reaction tests for 12 clinical samples collected from 2 Chinese Milu at Beijing Milu Ecological Research Center and 6 spiked samples from human blood and stool. Overall, the constructed *C. perfringens* detection methods, RAA-CRISPR/Cas12a-FL and RAA-CRISPR/Cas12a-LFS, have great potential as a novel detection scheme for the early diagnosis of *C. perfringens* infection in humans and animals.

## 1. Introduction

*Clostridium perfringens*, a Gram-positive and spore-forming bacterium, is an important human and animal enteric pathogen that can cause a wide diversity of diseases. *C. perfringens* is known for its ability to cause human gas gangrene as well as one of the most common causes of foodborne disease burden in the USA and European countries [1, 2]. In livestock and poultry, it can cause hemorrhagic bowel syndrome and enterotoxaemia in ruminants [3], hemorrhagic gastroenteritis in dogs and horses [4], and necrotic enteritis in poultry [5, 6]. In recent years, many cases of hemorrhagic enteritis caused by *C. perfringens* have also been reported in wild animals, especially in Chinese Milu (Père David's deer, *Elaphurus davidiensis*) [7–11]. Furthermore, hemorrhagic enteritis is characterized by acute onset, short course, and high fatality rate [9]. In view of these aspects, the rapid and sensitive detection of *C. perfringens* is crucial for the conservation of wildlife and keeping human and animal health.

Currently, the detection methods for *C. perfringens* mainly include the microbial culture-based technology [12, 13], antigen–antibody interaction-based technology [14, 15], and nucleic acid amplification-based technology (NAT) [16, 17]. A traditional culture-based method is recognized as the gold standard method for the diagnosis of *C. perfringens* infection [12, 13]; however, it is laborious and time-consuming. Immunological methods such as toxin neutralization assay and enzyme-linked immunosorbent assay have low diagnostic sensitivity [15, 18]. Molecular detection methods of *C. perfringens* have been applied, including polymerase chain reaction (PCR) assay and real-time PCR assay [16, 17]; however, these assays rely on specialized and expensive equipment and/or professional personnel, which limits their application in the field and resource-poor areas. Advances in isothermal amplification methods, including loop-mediated isothermal amplification (LAMP) and recombinase-aided amplification (RAA), have made it possible for NAT to on-site application [19, 20]. Unsurprisingly, LAMP and LAMP in combination with lateral flow strip (LFS) methods for the detection of *C. perfringens* in food and fecal samples have been developed [13, 21, 22], although these methods still have some defects, such as aerosol pollution-leaded false positive and the difficulty in primer design.

Recently, several seminal discoveries of clustered regularly interspaced short palindromic repeat (CRISPR)-associated proteins have made CRISPR/Cas system a favorite in the area of nucleic acid detection owing to its high simplicity, sensitivity, and specificity [23–25]. Several CRISPR/Cas-based diagnostic technologies have been developed using a unique group of Cas enzymes (Cas12a, Cas12b, and Cas13a) [23–26]. Cas12a (Cpf1), an RNA-guided DNA-targeting enzyme, recognizes DNA sequence as an activator and then cleaves nonspecific single-strand DNA reporter (termed collateral cleavage) [23]. Based on this property, CRISPR/Cas12a-based nucleic acid detection system combined with RAA or LAMP has been successfully applied to detect a variety of pathogens, such as SARS-CoV-2 [27], human papillomavirus [23], *Vibrio vulnificus* [28], *Aeromonas hydrophila* [29], *Escherichia coli* O157:H7 [30], and *Streptococcus aureus* [30].

**Figure 1:**
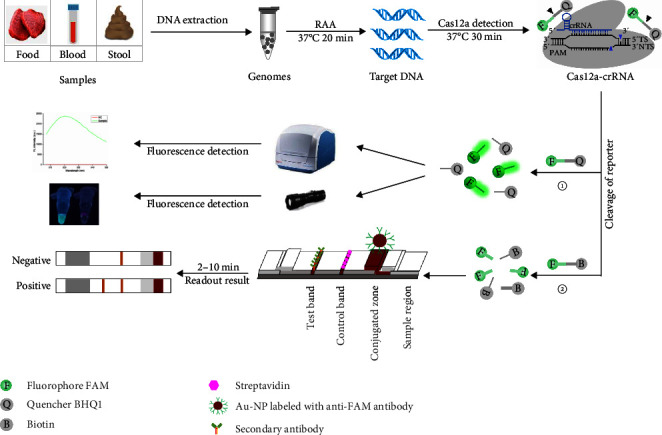
Workflow of RAA-CRISPR/Cas12a-based system in the detection of *C. perfringens* using ssDNA-FQ and ssDNA-FB reporters.

The pathogenic mechanism of *C. perfringens* is mainly attributable to its copious toxin production [31–33]. Based on the patterns of toxin (*α*-toxin, *β*-toxin, *ε*-toxin, *ι*-toxin, enterotoxin, and necrotic enteritis toxin B), *C. perfringens* can be classified into seven toxin types (A–G), and each toxin type can express *cpa*-encoded *α*-toxin and cause animal death [31, 32, 34]. In this study, based on RAA assay-assisted CRISPR/Cas12a system, two *C. perfringens* detection methods targeting the *cpa* gene, RAA-CRISPR/Cas12a-FL and RAA-CRISPR/Cas12a-LFS, were developed through reading fluorescence signal and LFS, respectively (Figure 1). These two methods are free of elaborate instruments and show high sensitivity in the detection of *C. perfringens* genomic DNA and high specificity in the detection of clinical Milu samples and spiked human samples, and the whole process can be completed in 1 hr. Thus, the developed RAA-CRISPR/Cas12a-FL and RAA-CRISPR/Cas12a-LFS methods may be the promising approach for on-site *C. perfringens* detection in samples from humans and animals.

## 2. Materials and Methods

### 2.1. Bacterial Strains and Genomic DNA Extraction

A total of 16 bacterial strains, including 11 reference strains and 5 isolation strains, were used in this study. Eleven reference strains were *C. perfringens* Type A (CVCC 2015), *C. perfringens* Type B (CVCC 54), *C. perfringens* Type C (CVCC 1153), *C. perfringens* Type D (CVCC 60201), *E. coli* (ATCC 25922), *S. aureus* (ATCC 25923), *Pseudomonas aeruginosa* (ATCC 27853), *Bacillus cereus* (ATCC 14579), *A. hydrophila* (ATCC 7966), *V. vulnificus* (ATCC 27562), and *Vibrio harvey* (ATCC 14126). Five isolation strains were *C. perfringens* strain MLa, *C. perfringens* strain MLb, *Salmonella typhimurium*, *Vibrio parahaemolyticus*, and *Edwardsiella piscicida*. *C. perfringens* reference strains were kindly gifted by China Veterinary Culture Collection Center. Two *C. perfringens* strains, MLa and MLb, were isolated from clinical samples of dead Père David's deer infected with *C. perfringens*. Ten non-*C. perfringens* bacteria mentioned above were employed as a negative control to evaluate the specificity of the two developed methods in the detection of *C. perfringens*.

*C. perfringens* strains were cultured in tryptose-sulfite-cycloserine medium under anaerobic conditions, while nontarget bacterial strains were inoculated into 2216E broth or Luria-Bertani medium. A MiniBEST Bacteria Genomic DNA Extraction Kit Ver.3.0 (9763; TaKaRa Bio Inc., Japan) was employed to extract genomic DNA from bacterial cultures.

### 2.2. RAA Assay

The RAA primer sequences specific for *C. perfringens cpa* gene were designed using Primer 5 software (Table S1). The bacterial genomic DNA was used as the template of the RAA reaction according to the RAA basic kit (Jiangsu Qitian Gene Biotechnology, China). Briefly, a total 50 *μ*L RAA reaction system containing 25 *µ*L of buffer V, 2 *µ*L each of forward and reverse primers, 2 *µ*L of DNA template, 16.5 *µ*L of purified water, and 2.5 *µ*L of magnesium acetate was prepared, and the mixture was then incubated at 37°C. The products of RAA were finally analyzed with agarose gel or used as the target of the CRISPR/Cas12a system.

### 2.3. RAA-CRISPR/Cas12a-FL Assay and RAA-CRISPR/Cas12a-LFS Assay

**Figure 2:**
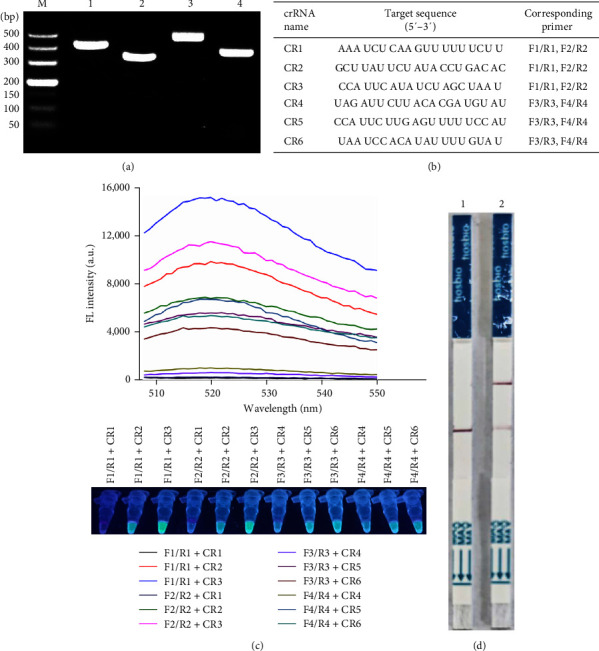
Feasibility verification of the RAA-CRISPR/Cas12a-based *C. perfringens* detection methods. (a) Efficiency evaluation of designed four RAA primer sets using gel electrophoresis. M, 500 DNA marker; lanes 1–4, RAA product of primer set 1, 2, 3, and 4, respectively. (b) Target sequences and their corresponding primer sets of six crRNAs were used in this study. (c) Screening the optimal combination of primer set and crRNA using RAA-CRISPR/Cas12a-FL assay through a multifunctional microplate reader (upper) or a UV flashlight (below). (d) Verifying the feasibility of the RAA-CRISPR/Cas12a-LFS method using the F1/R1-CR3 combination. 1, RAA template is H_2_O; 2, RAA template is *C. perfringens* genomic DNA.

The CRISPR-derived RNA (crRNA), fluorophore quencher-labeled single-stranded DNA reporter (5′-/6-FAM/TTATT/BHQ1/-3′; ssDNA-FQ), and fluorophore biotin-labeled single-stranded DNA reporter (5′-/6-FAM/TTATT/Bio/-3′; ssDNA-FB) were designed as described by Xiao et al. [28] and Broughton et al. [27] and synthesized by Sangon Biotech (Shanghai, China). LFS were purchased from Tiosbio (JY0301; Beijing, China). The designed crRNA sequences are shown in Figure 2(b). RAA-CRISPR/Cas12a-FL detection assay was performed as follows: 10 *μ*L of 200 nM Cas12a (New England Biolabs, USA) and 10 *μ*L 200 nM crRNA were preincubated at 37°C for 20 min. After this, 10 *μ*L of 500 nM ssDNA-FQ reporters and 2 *µ*L of RAA products were added to the above reaction tube and incubated at 37°C for 30 min. UV flashlight or a multifunctional microplate reader (*λ*_ex_: 485 nm and *λ*_em_: 520 nm) was used for fluorescence detection. As for the RAA-CRISPR/Cas12a-LFS assay, ssDNA-FB reporter was used, and the result would be determined by the colorimetric signal of the LFS at the end of the reaction.

Optimization of RAA reaction time and Cas12a cleavage time was conducted using RAA-CRISPR/Cas12a-FL assays. In terms of the sensitivity of RAA-CRISPR/Cas12a-based *C. perfringens* detection methods, serial 10-fold dilutions ranging from 10^0^ to 10^6^ copies/*μ*L of the *C. perfringens* genomic DNA were used as templates of RAA reaction. The specificity of two *C. perfringens* detection methods was determined by evaluating the cross-reactivity with nontarget bacteria.

### 2.4. Real-Time PCR Assay

Standard qPCR assay [16] was used to detect *C. perfringens* according to the instructions of the CFX96 real-time PCR detection system (Bio-Rad, the United States). Briefly, the qPCR reaction mixtures contained 10 *μ*L of ChamQ SYBR qPCR Master Mix (Vazyme Biotech, China), 0.5 *μ*L of forward and reverse primers, 2 *μ*L of DNA, and 7 *μ*L of ddH_2_O. The amplification conditions were 95°C for 30 s, followed by 39 cycles of 95°C for 5 s and 60°C for 30 s.

### 2.5. Clinical and Spiked Sample Analysis

Clinical samples were collected from the jejunum, lung, heart, liver, spleen, and kidney of two dead Milu, and one of them was diagnosed with *C. perfringens* infection. Blood and fecal samples were collected from three healthy volunteers, and 100 *μ*L of blood or 200 mg of stool was added into the tube containing 1 × 10^3^ CFU (colony-forming units) of *C. perfringens* to prepare spiked samples. Then, genomic DNA was extracted using TIANamp Genomic DNA Kit and Stool DNA Kit (Tiangen Biotech, Beijing, China), and 2 *μ*L of genomic DNA was used as the template for RAA-CRISPR/Cas12a-based *C. perfringens* detection system.

### 2.6. Statistical Analysis

Statistical analysis was performed using SPSS 13.0 software (SPSS Inc., Chicago, IL, USA). The data were analyzed by Student's *t*-test. *p* < 0.05 (indicated by  ^*∗*^) was considered statistically significant.

## 3. Results

### 3.1. Establishing the RAA-CRISPR/Cas12a-Based *C. perfringens* Detection Methods

To detect *C. perfringens* rapidly, sensitively, and specifically, an RAA-CRISPR/Cas12a-based *C. perfringens* detection system was generated, as shown in Figure 1. First, four RAA primer sets were designed, and evaluated their efficiency according to the intensity of RAA product band using the gel electrophoresis. The results showed that all the RAA product bands were clearly distinguishable (Figure 2(a)). According to the design principle of crRNA [28], six crRNAs targeting the conserved region of the RAA amplicon (CR1, CR2, and CR3 targeting the F1/R1 and F2/R2 amplicons; CR4, CR5, and CR6 targeting the F3/R3 and F4/R4 amplicons) were designed (Figure 2(b)).

To investigate the efficiency of each combination of RAA primer set and crRNA and verify the feasibility of RAA-CRISPR/Cas12a-based methods, different combinations of RAA primer set and crRNA were screened using *C. perfringens* (CVCC 2015) genomic DNA as the template of RAA assay. As shown in Figure 2(c), the combination of F1/R1 and CR3 triggered a stronger fluorescence signal than other combinations. Meanwhile, the feasibility of RAA-CRISPR/Cas12a-LFS method in the detection of *C. perfringens* using F1/R1-CR3 combination was also verified, and the results showed that the test band only appeared in the *C. perfringens* genomic DNA group (Figure 2(d)). Therefore, CR3 and its corresponding primer set, F1/R1, were selected as the optimal crRNA and primer set and used to perform the follow-up assays.

### 3.2. Optimizing RAA Reaction Time and Cas12a Cleavage Time

**Figure 3:**
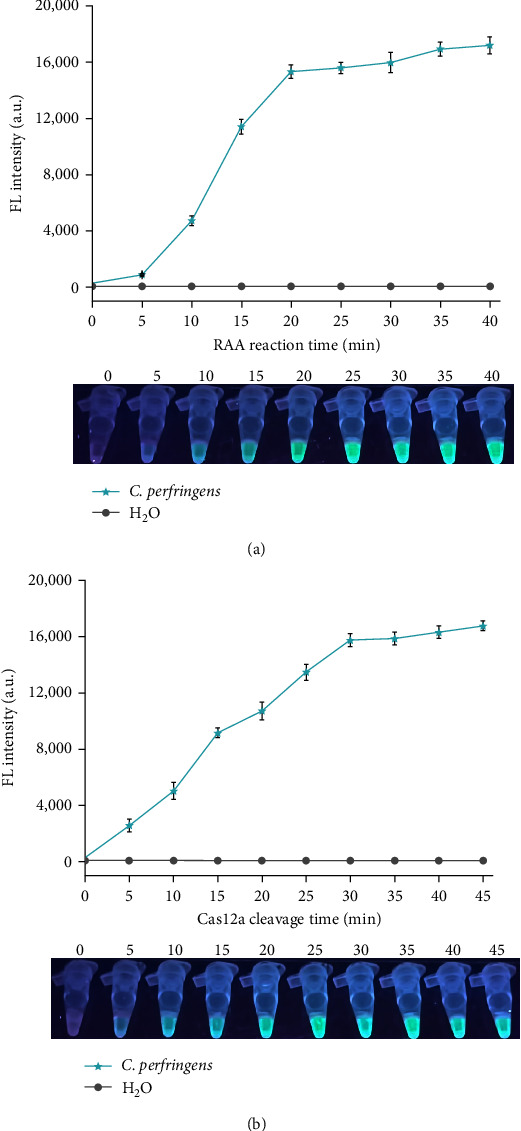
Optimization of the assay time of RAA-CRISPR/Cas12a system in the detection of *C. perfringens*. The RAA-CRISPR/Cas12a-FL assays were conducted using 1 × 10^5^ copies/*μ*L of *C. perfringens* genomic DNA as the template to obtain the optimal time of RAA reaction (a) and Cas12a cleavage (b), and the fluorescence signals were detected with a multifunctional microplate reader (upper) or a UV flashlight (below). *n* = 3 technical replicates; bars represent mean ± SEM.

A time-course study of the RAA reaction and Cas12a cleavage was conducted to optimize the assay time and achieve an ideal assay performance. To obtain an optimal RAA reaction time, different points of RAA reaction time (0, 5, 10, 15, 20, 25, 30, 35, and 40 min) were tested, while 45 min was chosen as the Cas12a cleavage time. As shown in Figure 3(a), although the fluorescence signal was on the increase with time, fluorescence intensity reached a plateau after 20 min; hence, 20 min was selected as the optimal time of the RAA reaction. Moreover, as for Cas12a cleavage time, fluorescence signals of different points of Cas12a cleavage time (0, 5, 10, 15, 20, 25, 30, 35, 40, and 45 min) were detected, and the results showed that the Cas12a cleavage efficacy was essentially completed within 30 min (Figure 3(b)). Consequently, the optimal detection time of the RAA-CRISPR/Cas12a system was considered as 50 min (20 min for RAA reaction and 30 min for Cas12a cleavage), which was adopted for subsequent experiments.

### 3.3. Sensitivity of RAA-CRISPR/Cas12a-Based Methods for Detecting *C. perfringens*

**Figure 4:**
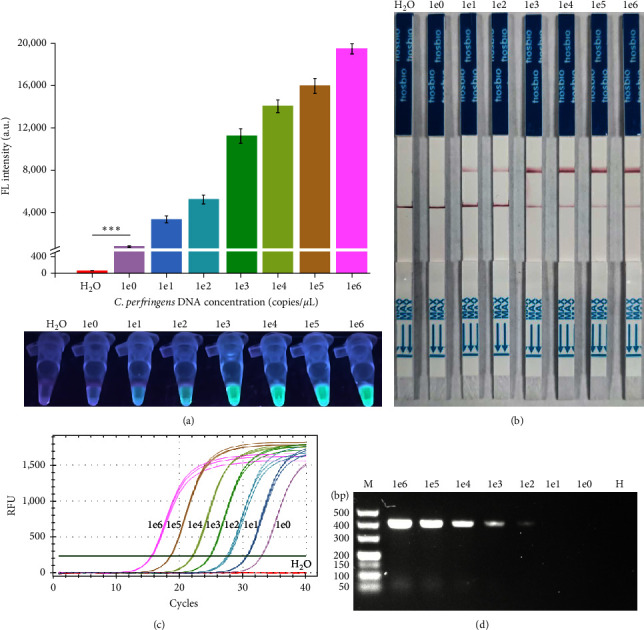
Evaluation of the sensitivity of RAA-CRISPR/Cas12a-based methods for detecting *C. perfringens*. The sensitivity of RAA-CRISPR/Cas12a-FL (a), RAA-CRISPR/Cas12a-LFS (b), qPCR (c), and RAA (d) assays was assessed in the detection of *C. perfringens*. The templates of these assays were 1 × 10^0^ to 1 × 10^6^ copies/*μ*L of *C. perfringens* genomic DNA and nuclease-free H_2_O. Data are one representative of three experiments. (a) *n* = 3 technical replicates; two-tailed Student's *t* test;  ^*∗∗∗*^*p* < 0.001, experimental group versus H_2_O group (only shown the 1e0 vs. H_2_O); bars represent mean ± SEM.

To investigate the sensitivity of the RAA-CRISPR/Cas12a-FL and RAA-CRISPR/Cas12a-LFS methods in the detection of *C. perfringens*, the *C. perfringens* genomic DNA (ranging from 10^0^ to 10^6^ copies/*μ*L) were serially diluted for the evaluation of their limit of detection (LOD). About 2 *μ*L of genomic DNA and an equal volume of nuclease-free H_2_O were used as templates to perform the RAA-CRISPR-based assays. As shown in Figure 4(a), fluorescence signals could be generated by all the *C. perfringens* DNA samples but not by H_2_O according to the results of RAA-CRISPR/Cas12a-FL assays, suggesting that the LOD of this method for the detection of *C. perfringens* was 2 copies/reaction. As for the other RAA-CRISPR/Cas12a-based method, RAA-CRISPR/Cas12a-LFS, which is more convenient for on-site detection of *C. perfringens*, the results showed that only these 10^1^ to 10^6^ copies/*μ*L *C. perfringens* DNA samples could trigger the appearance of the test band in the LFS (Figure 4(b)), suggesting that the LOD of this method was 20 copies/reaction.

To evaluate the sensitivity difference between the developed methods and other methods in the detection of *C. perfringens*, the LOD of qPCR assay and RAA assay in the detection of *C. perfringens* was assessed. As shown in Figure 4(c), qPCR assay could detect all the *C. perfringens* DNA samples, indicating that the LOD of the RAA-CRISPR/Cas12a-FL method is the same as that of qPCR. As for the RAA assay that was conducted using the same condition as the RAA-CRISPR/Cas12a system, the LOD of the RAA assay was 200 copies/reaction (Figure 4(d)), which is significantly lower than that of the RAA-CRISPR/Cas12a-FL and RAA-CRISPR/Cas12a-LFS. Therefore, these two developed methods, RAA-CRISPR/Cas12a-FL and RAA-CRISPR/Cas12a-LFS showed high sensitivity in the detection of *C. perfringens*.

### 3.4. Specificity of RAA-CRISPR/Cas12a-Based Methods for Detecting *C. perfringens*

**Figure 5:**
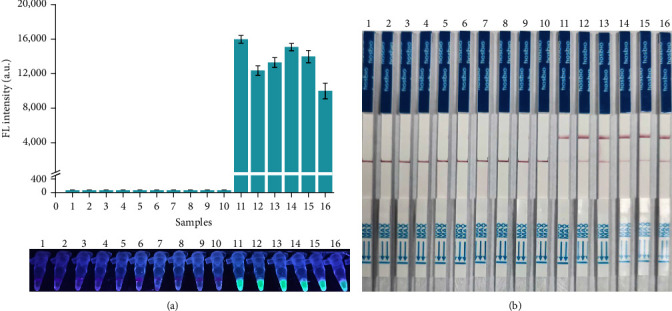
Evaluation of the specificity of RAA-CRISPR/Cas12a-based methods for detecting *C. perfringens*. Six *C. perfringens* strains and 10 other related zoonotic bacterial pathogens were used to evaluate the specificity of RAA-CRISPR/Cas12a-FL and RAA-CRISPR/Cas12a-LFS methods in the detection of *C. perfringens*, and the fluorescence signals were detected with a multifunctional microplate reader (upper) or a UV flashlight (below). 1, *Salmonella typhimurium*; 2, *Vibrio parahaemolyticus*; 3, *Edwardsiella piscicida*; 4, *Escherichia coli*; 5, *Staphylococcus aureus*; 6, *Pseudomonas aeruginosa*; 7, *Bacillus cereus*; 8, *Aeromonas hydrophila*; 9, *Vibrio vulnificus*; 10, *Vibrio harvey*; 11, *C. perfringens* strain MLa; 12, *C. perfringens* strain MLb; 13, *C. perfringens* Type A; 14, *C. perfringens* Type B; 15, *C. perfringens* Type C; 16, *C. perfringens* Type D. *n* = 3 technical replicates; bars represent mean ± SEM.

To investigate the specificity of the RAA-CRISPR/Cas12a-FL and RAA-CRISPR/Cas12a-LFS methods in the detection of *C. perfringens*, 6 *C. perfringens* strains, and 10 other related zoonotic bacterial pathogens—that were used as a control in this test—were selected as the detection samples of these 2 methods. As shown in Figure 5, the tubes with *C. perfringens* genomic DNA generated a strong fluorescence signal (Figure 5(a)) and colorimetric signal in the test band of LFS (Figure 5(b)), whereas no signals were observed from non-*C. perfringens* strains. These data clearly demonstrated that the RAA-CRISPR/Cas12a-based methods showed high specificity in the detection of *C. perfringens*.

### 3.5. Detection of *C. perfringens* in Clinical and Spiked Samples with RAA-CRISPR/Cas12a-Based Methods

**Figure 6:**
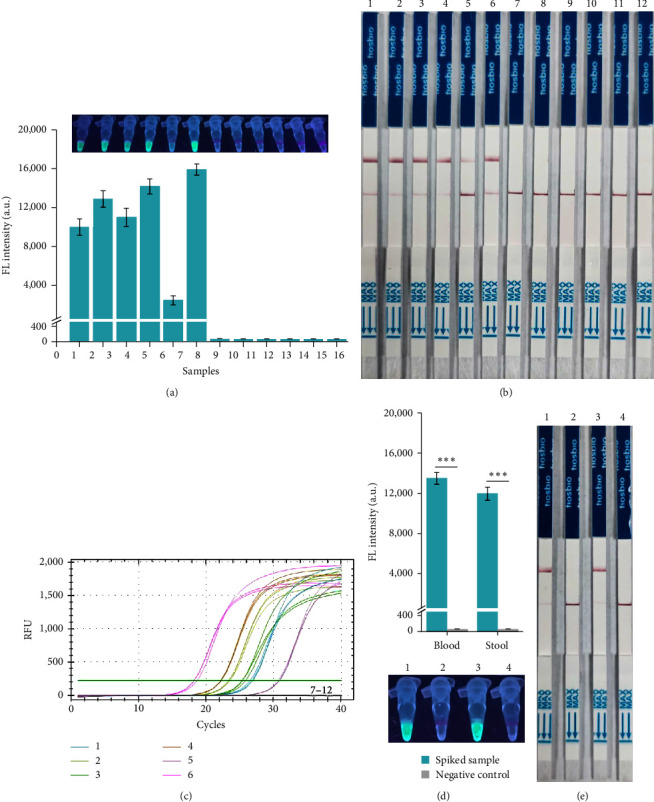
Evaluation of the practicability of RAA-CRISPR/Cas12a-based methods for detecting *C. perfringens* in clinical and spiked samples. Genomic DNAs were extracted from the tissues of two abnormal death Milu and then were detected using RAA-CRISPR/Cas12a-FL (a), RAA-CRISPR/Cas12a-LFS (b), and qPCR (c) assays. 1–6 were lung, heart, spleen, kidney, liver, and jejunum, respectively, collected from the Milu dying of *C. perfringens*; 7–12 were lung, heart, spleen, kidney, liver, and jejunum, respectively, collected from the Milu dying of non-*C. perfringens* pathogen. (d and e) Human blood and fecal samples were collected and used to evaluate the practicability of RAA-CRISPR/Cas12a-based methods in the diagnosis of patients infected with *C. perfringens*. Blood samples and fecal samples were contaminated with 1 × 10^3^ CFU *C. perfringens*, and then the spiked samples and normal samples were detected using RAA-CRISPR/Cas12a-FL (d) and RAA-CRISPR/Cas12a-LFS (e) methods. 1, spiked blood sample; 2, normal blood sample; 3, spiked fecal sample; 4, normal blood sample. *n* = 3 technical replicates; two-tailed Student's *t* test;  ^*∗∗∗*^*p* < 0.001; bars represent mean ± SEM.

According to the above results, two rapid RAA-CRISPR/Cas12a-based *C. perfringens* detection methods with high sensitivity and specificity have been established. Finally, the performance of these two methods in the detection of clinical and spiked samples was evaluated. Twelve tissue samples, including the lung, heart, liver, spleen, kidney, and jejunum from two abnormal death Milu (one of which was diagnosed as *C. perfringens* infection) were used to retrospectively test *C. perfringens*. As shown in Figures 6(a) and 6(b), either the RAA-CRISPR/Cas12a-FL method (Figure 6(a)) or RAA-CRISPR/Cas12a-LFS method (Figure 6(b)) could only detect all six *C. perfringens*-infected samples, which was consistent with the results of qPCR assay (Figure 6(c)). These findings were in line with the results of the traditional culture-based method (data not shown). These results demonstrated that these developed methods could resist the influence of Milu genomic DNA and be used to detect *C. perfringens*-infected Milu.

For spiked samples, blood and fecal samples collected from three volunteers and contaminated with low levels of *C. perfringens* (1 × 10^3^ CFU) were detected with RAA-CRISPR/Cas12a-FL (Figure 6(d)) and RAA-CRISPR/Cas12a-LFS (Figure 6(e)) methods. These results showed that only the spiked samples could be detected and demonstrated that the developed methods could also be used to diagnose patients infected with *C. perfringens*.

Taken together, the developed RAA-CRISPR/Cas12a-based methods presented a significant advantage over existing methods, allowing rapid, sensitive, specific, and instrument-free detection of *C. perfringens* in clinical Milu samples and spiked human samples.

## 4. Discussion

*C. perfringens* is responsible for many histotoxic and enterotoxic infections in humans and many animals [32]; for example, hemorrhagic enteritis caused by *C. perfringens* has become one of the most important diseases of Milu [9, 35, 36]. Therefore, strengthening the research on the key technology for the prevention and control of this bacteria has been gaining widespread attention. Currently, the detection methods of *C. perfringens* mainly rely on conventional culture technology and NAT (real-time PCR, PCR, and LAMP) [13, 16, 17, 21]. Although the real-time PCR method has been widely validated and is considered the gold standard test for *C. perfringens*, it still has some shortcomings because of the complicated operation and sophisticated equipment. Other molecular detection methods, PCR and LAMP, depending on the gel electrophoresis analysis and lateral flow dipstick, show lower sensitivity than the real-time PCR method [13, 21]. In addition, fully equipped diagnostic laboratories are usually far from Wild Animal Park and breeding bases for endangered wild animals, which is difficult to conduct the above methods in these places and may lead to delayed diagnosis. To address these shortcomings, two novel *C. perfringens* detection methods, RAA-CRISPR/Cas12a-FL and RAA-CRISPR/Cas12a-LFS (Figure 1), were developed, which can be used for on-site *C. perfringens* detection with time-saving (Figure 3), instrument-free, high sensitivity (Figure 4), and high specificity (Figure 5). To our knowledge, this research is the first time to report the RAA-CRISPR-Cas12a-based methods for the detection of *C. perfringens*.

It is well known that the pathogenicity of *C. perfringens* is determined by multiple toxins [3]. All types of *C. perfringens* isolates produce cpa gene-encoded *α*-toxin, which possesses phospholipase C and sphingomyelinase activity and plays key roles in the pathogenicity of *C. perfringens* [31, 32, 34]. The feature of *cpa* gene exhibits the high sequence conservation and species specificity and has been widely used as a target gene to identify *C. perfringens* [13, 21, 22]. Therefore, in this study, *cpa* gene was selected for detecting *C. perfringens*. The published *cpa* sequences in GenBank were downloaded and aligned, and then RAA primers and crRNAs were designed according to the conserved region (Table S1; Figure 2(b)). Twelve combinations of RAA primer set and crRNA were obtained, and upon screening using RAA-CRISPR/Cas12a-FL assay, one combination, F1/R1-CR3, exhibited the highest activity among these 12 combinations (Figure 2(c)). Meanwhile, the activity of the F1/R1-CR3 combination was also verified using RAA-CRISPR/Cas12a-LFS method (Figure 2(d)). The validity of the F1/R1-CR3 combination was further confirmed in the specificity test of two RAA-CRISPR/Cas12a-based methods, which shows that only six *C. perfringens* strains could be detected using the established methods, RAA-CRISPR/Cas12a-FL (Figure 5(a)) and RAA-CRISPR/Cas12a-LFS (Figure 5(b)).

Previously, RAA assay coupled with CRISPR/Cas12a system has been established for pathogen detection and showed high sensitivity [28, 29]. In this study, these results demonstrated that RAA-CRISPR-Cas12a-FL and RAA-CRISPR/Cas12a-LFS methods based on fluorescence signal and colorimetric signal detected the *C. perfringens* genomic DNA at a sensitivity level of 2 copies/reaction (Figure 4(a)) and 20 copies/reaction (Figure 4(b)), respectively. The sensitivity of these two methods was nearly equal to that of real-time PCR but higher than RAA assay (Figure 4(d)) and LAMP assay [13, 21]. Because *C. perfringens* could cause diseases in Milu and humans [32, 35], the practicability of these proposed methods in clinical Milu samples and spiked human samples was investigated. Using RAA-CRISPR/Cas12a-FL and RAA-CRISPR/Cas12a-LFS methods, *C. perfringens* detection from clinical and spiked samples could be completed in 1 hr. Although the detection results between RAA-CRISPR-Cas12a-based methods and real-time PCR were 100% consistent, the RAA-CRISPR-Cas12a-based methods spent less time.

In summary, the current study first presented RAA-CRISPR/Cas12a-FL and RAA-CRISPR/Cas12a-LFS methods for *C. perfringens* detection with time-saving, instrument-free, and high sensitivity. They may serve as an alternative scheme for the rapid diagnosis of patients and animals infected with *C. perfringens* to prevent its spread at an early stage.

## Data Availability

The data that support the findings of this study are available from the corresponding authors upon reasonable request.

## Supplementary Materials

Supplementary Materials Table S1: designed RAA primer sequences specific for *C. perfringens cpa* gene.
