# Supplementary material for: Detection of Clostridium perfringens Using Novel Methods Based on Recombinase-Aided Amplification Assay-Assisted CRISPR/Cas12a System
Source: Transbound Emerg Dis. 2023 Nov 14;2023:6667618. doi: 10.1155/2023/6667618 (PMC12016868; doi:10.1155/2023/6667618)
Supplement: Supplementary Materials — Table S1: designed RAA primer sequences specific for C. perfringens cpa gene. [file 6667618.f1.doc]

**Table S1**. Designed RAA primer sequences specific for *C. perfringens cpa* gene.

| Primer Name | Sequence (5’-3’) |
| --- | --- |
| F1 | TAAAGTCTACGCTTGGGATGGAAAGATTGA |
| R1 | TATATCTCCAAAATAGTGCATAGCCTCTCC |
| F2 | CTGTCCAAAAATGAACCAGAAAGTGTAAGA |
| R2 | TATATCTCCAAAATAGTGCATAGCCTCTCC |
| F3 | AACAGGAAAATCAATATACTATAGTCATGCT |
| R3 | TTTGCTATTATCTTTATGTTTTCTGGCTTA |
| F4 | GAACAGCGGGATATATTTATAGATTCTTAC |
| R4 | TTTGCTATTATCTTTATGTTTTCTGGCTTA |
